# Identification and Protective Efficacy of *Eimeria tenella* Rhoptry Kinase Family Protein 17

**DOI:** 10.3390/ani12050556

**Published:** 2022-02-23

**Authors:** Xiaoxin Liu, Bingjin Mu, Wenbin Zheng, Yijing Meng, Linmei Yu, Wenwei Gao, Xingquan Zhu, Qing Liu

**Affiliations:** 1 College of Veterinary Medicine, Shanxi Agricultural University, Jinzhong 030801, China; vetlxx@163.com (X.L.); mbj142301@163.com (B.M.); wenbinzheng1@126.com (W.Z.); mmmmeng_y@163.com (Y.M.); ylm0719@163.com (L.Y.); sxndgaowenwei@163.com (W.G.); xingquanzhu1@hotmail.com (X.Z.); 2 Key Laboratory of Veterinary Public Health of Higher Education of Yunnan Province, College of Veterinary Medicine, Yunnan Agricultural University, Kunming 650201, China

**Keywords:** *Eimeria tenella*, rhoptry kinase family protein 17, merozoite, protective efficacy

## Abstract

**Simple Summary:**

Approximately 8000 genes of *Eimeria tenella* have been found by genome sequencing, whereas very few data are currently available regarding *E. tenella* rhoptry kinase family proteins. In this study, the coding sequence of the rhoptry kinase family protein 17 of *E. tenella* (EtROP17) was cloned and expressed in *Escherichia coli*, and then the protective efficacy of the recombinant EtROP17 (rEtROP17) was assessed in chickens. Sequence analysis showed that a single base difference at position 1901 of the ROP17 of the SD-01 strain was observed compared with that of the Houghton strain. EtROP17 was expressed in the merozoite stage of *E. tenella* and may be a potential vaccine candidate against coccidiosis.

**Abstract:**

*Eimeria tenella* encodes a genome of approximately 8000 genes. To date, however, very few data are available regarding *E. tenella* rhoptry kinase family proteins. In the present study, the gene fragment encoding the mature peptide of the rhoptry kinase family protein 17 of *E. tenella* (EtROP17) was amplified by PCR and expressed in *E. coli.* Then, we generated polyclonal antibodies that recognize EtROP17 and investigated the expression of EtROP17 in the merozoite stage of *E. tenella* by immunofluorescent staining and Western blot analysis. Meanwhile, the protective efficacy of rEtROP17 against *E. tenella* was evaluated in chickens. Sequencing analysis showed that a single base difference at sequence position 1901 was observed between the SD-01 strain and the Houghton strain. EtROP17 was expressed in the merozoite stage of *E. tenella.* The results of the animal challenge experiments demonstrated that vaccination with rEtROP17 significantly reduced cecal lesions and oocyst outputs compared with the challenged control group. Our findings indicate that EtROP17 could serve as a potential candidate for developing a new vaccine against *E. tenella*.

## 1. Introduction

Coccidiosis, which is caused by several intracellular intestinal parasites of the genus *Eimeria*, is the most severe parasitic disease responsible for substantial economic losses to the poultry industry all around the world [1,2,3]. Annual global economic losses due to coccidiosis are estimated to be USD 3 billion [4]. Conventional strategies used to control the disease depend heavily on chemoprophylaxis [5,6]. Meanwhile, live anticoccidial vaccines are used commercially for the control of avian coccidiosis [6]. However, the use of live anticoccidial vaccines or anti-parasitic drugs has disadvantages, such as the occurrence of drug resistance, complex production processes, and potential pathogenicity [7,8]. Hence, the development of novel control measures such as subunit vaccines has attracted widespread attention [9].

*Eimeria tenella* is an important species owing to its association with many coccidiosis outbreaks [10]. Approximately 8000 genes of this parasite have been identified by genome sequencing [11]. To date, however, the expression of several proteins, such as rhoptry kinase family proteins, in different developmental phases of the parasite is unknown [12]. *E. tenella* rhoptry kinase family protein 17 (EtROP17) has been identified in the rhoptry proteome of *E. tenella* sporozoites, whereas its expression in other developmental phases of the parasite remains unknown [13].

In recent years, a number of *E. tenella* antigens have been tested as candidate antigens for subunit or DNA vaccines, such as surface antigen 16 (SAG16), SAG22, gametocyte 22 antigen, and silent information regulator 2 [9,14,15]. Currently, the protective efficacy of several proteins has been not evaluated, such as EtROP17.

In the present study, the recombinant protein EtROP17 (rEtROP17) was expressed in a prokaryotic expression system. Then, we generated polyclonal antibodies that recognized EtROP17 and investigated the expression of EtROP17 in the merozoite phase of *E. tenella*. Furthermore, its protective efficacy against *E. tenella* was determined in a chicken challenge model.

## 2. Materials and Methods

### 2.1. Parasites and Animals

Sporulated oocysts of the SD-01 strain of *E. tenella* were stored in 2.5% potassium dichromate and propagated in *Eimeria*-free chicks as previously described [16]. One-day-old Hy-Line layer chicks were reared in isolators under *Eimeria*-free conditions. BALB/c female mice aged four to five weeks were purchased from the Animal Center of Shanxi Medical University (Shanxi, China).

### 2.2. Amplification and Recombinant Expression of EtROP17

**Figure 1:**
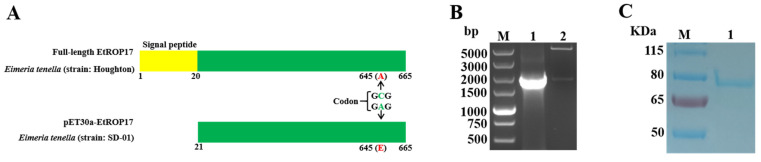
(**A**) The region of EtROP17 used for expression: the C to A transition at nucleotide 1901 converted the amino acid alanine (position 645) to amino acid glutamic acid. (**B**) Lane M: DL5000 DNA marker; lane 1: the gene fragment of EtROP17 amplified from *E. tenella* cDNA; lane 2: the recombinant plasmid was identified by digestion. (**C**) Lane 1: SDS-PAGE analysis of purified rEtROP17.

Total RNA was isolated from the *E. tenella* sporozoites by using TRI Reagent^®^ (Sigma-Aldrich, St. Louis, MO, USA), which was reverse-transcribed into cDNA using a PrimeScript™ 1st Strand cDNA Synthesis Kit (Takara, Dalian, China). The gene fragment encoding the mature peptide of EtROP17 (from aa21 to aa665) (Figure 1A) was amplified from the cDNA by PCR using two gene-specific primers: EtROP17-F (5′-GGATCCGTCGACTGTCATCGGGCC-3′) and EtROP17-R (5′-GGGGAATTCCTAAACTGAGTTAGTTTCAGACGAG-3′). To construct the recombinant plasmid, the amplified specific fragment was purified and ligated into the BamHI/EcoRI sites of a pET30a vector (designated pET30a-EtROP17). The recombinant vector was verified by endonuclease digestion and DNA sequencing (Tsingke Biotechnology Co., Ltd., Beijing, China). The obtained DNA sequences were compared using the Basic Local Alignment Search Tool (BLAST) (https://blast.ncbi.nlm.nih.gov/Blast.cgi (accessed on 27 June 2021). rEtROP17 was expressed in *Escherichia coli* BL21 (DE3) and purified using the His60 Ni Gravity Columns (Takara, Dalian, China).

### 2.3. Generation of Antisera

The immunized serum against *E. tenella* was prepared according to a previous study [17]. Briefly, chickens reared in *Eimeria*-free conditions until 2 weeks of age were infected orally with 1 × 10^4^ sporulated oocysts of the SD-01 strain per chicken. Three days after the first challenge, four additional inoculations at 3 day intervals were conducted with 5000 sporulated oocysts per chicken. Five weeks after the final challenge, blood samples were collected by heart puncture. Antiserum was obtained by centrifugation and stored at −80 °C until use. Serum collected from unchallenged chickens was used as the normal chicken serum.

The purified rEtROP17 was used as an antigen for generating anti-rEtROP17 serum. Mice were immunized intraperitoneally with the purified rEtROP17 protein (50 μg/mouse) emulsified in Freund’s complete adjuvant (Sigma-Aldrich, St. Louis, MO, USA). Subsequently, two additional immunizations at 2 week intervals were administered at the same dose with Freund’s incomplete adjuvant (Sigma-Aldrich, St. Louis, MO, USA). Ten days after the last immunization, blood samples were collected and used for the generation of anti-rEtROP17 serum.

### 2.4. Immunoblot Analysis of rEtROP17 and Native EtROP17 Protein

Following resolution by 10% SDS-PAGE, each purified protein sample was transferred to polyvinylidene fluoride (PVDF) membranes (Millipore, Bedford, MA, USA) using eBlot™ L1 (GenScript, Nanjing, China). The membranes were separately incubated at 37 °C for 2 h with mouse anti-His tag monoclonal antibody (CWBIO, Beijing, China), chicken anti-*E. tenella* serum, and normal chicken serum. After washing with TBST buffer, the membranes were separately incubated at 37 °C for 1 h with the corresponding secondary antibodies: peroxidase-conjugated goat anti-mouse IgG or HRP-conjugated goat anti-chicken IgY (Abcam, Cambridge, UK). The binding of secondary antibodies was revealed by the enhanced HRP-DAB chromogenic substrate kit (TIANGEN, Beijing, China).

Protein extracts of merozoites were obtained by using RIPA lysis buffer (Beyotime, Nantong, China). Western blot analysis was carried out using mouse anti-rEtROP17 serum as the primary antibody. Goat anti-mouse IgG conjugated with horseradish peroxidase (Abcam, Cambridge, UK) was used as a secondary antibody. The bound antibody was detected using protein enhanced chemiluminescent (ECL) reagent (Thermo Scientific, Waltham, MA, USA). The slide stained with the serum before immunization as the primary antibody was used as the control.

### 2.5. Immunofluorescence Analysis of Native EtROP17 Expression

An immunofluorescence technique was used to detect the expression of EtROP17 in merozoites, as previously described [18]. Merozoites were smeared on a glass slide and air-dried before fixation. Each slide was fixed with 2% paraformaldehyde for 10 min at room temperature, and then permeabilized with 0.1% Triton X-100 in PBS for 10 min. Mouse anti-rEtROP17 serum (dilution of 1:250) was used as the primary antibody. The secondary antibody was FITC-conjugated goat anti-mouse IgG (Abcam, Cambridge, UK) (dilution of 1:5000). The samples were visualized with a Nikon fluorescence microscope (Nikon, Tokyo, Japan).

### 2.6. Immunization and Challenge Infection

One-week-old chickens were weighed and randomly placed into 4 groups, each consisting of 12 birds [9]. The experimental design of immunizations and challenges is shown in Table 1. The experimental group was vaccinated subcutaneously with 50 μg or 100 μg of purified rEtROP17 emulsified with Montanide ISA 71 adjuvant (3:7). Chickens in the challenged control and unchallenged control groups were injected subcutaneously with the same B buffer (100 mM NaH_2_PO_4_, 10 mM Tris-HCI) emulsified with the same adjuvant. One week after the first vaccination, a booster vaccination was carried out as described above. All chickens, except those in the unchallenged control group, were infected orally with 10,000 freshly sporulated oocysts of the SD-01 strain. The unchallenged control group was given PBS orally.

### 2.7. Evaluation of Protective Efficacy

The response to infection of chickens in each group was assessed on the basis of body weight gain, lesion score, and oocyst shedding [19]. Body weight gain was determined by weighing the chickens on Day 8 post-challenge and subtracting the body weight on Day 0 post-challenge. Intestinal lesions were scored by using the system described by Johnson and Reid [20]. Fecal samples were collected at Day 6–8 post-challenge, and oocysts per gram were counted using the McMaster’s counting technique.

### 2.8. Determination of Serum Antibody Levels

Serum levels of anti-EtROP17 antibodies were examined by ELISA as described elsewhere [8]. Briefly, the 96-well plate was coated with purified rEtROP17 and incubated overnight at 4 °C. Following washing thrice with PBST, the plate was blocked with 5% skimmed milk in PBST. After washing thrice, wells were incubated with serum samples for 2 h at 37 °C. Bound antibodies were examined by incubation for 60 min with HRP-conjugated goat anti-chicken IgY antibody and 3, 3, 5, 5-tetramethylbenzidine (TMB). The reaction was stopped by using 1 M H_2_SO_4_, followed by measuring the absorbance at 450 nm with a microplate spectrophotometer.

### 2.9. Statistical Analysis

Statistical analysis was performed by using a one-way ANOVA Duncan test in the SPSS statistical software (SPSS 26, SPSS Inc., Chicago, IL, USA). Differences between groups at *p*-values smaller than 0.05 were considered to be significantly different.

## 3. Results

### 3.1. Amplification, Expression, and Purification

As shown in Figure 1B, the 1938-bp fragment of EtROP17 was successfully amplified. The resultant plasmid was checked and confirmed by restriction enzyme digestion. Sequencing analysis showed a single base difference at sequence position 1901 between the SD-01 strain and the Houghton strain. The C to A transition converted the amino acid alanine (position 645) to amino acid glutamic acid (Figure 1A). SDS-PAGE analysis showed that EtROP17 was successfully expressed in the prokaryotic expression system. The molecular weight of the fusion protein was estimated to be 78 KDa (Figure 1C), which is consistent with the size calculated on the basis of the presumptive amino acid sequence.

### 3.2. Identification and Immunoreactivity of the Recombinant Protein

**Figure 2:**
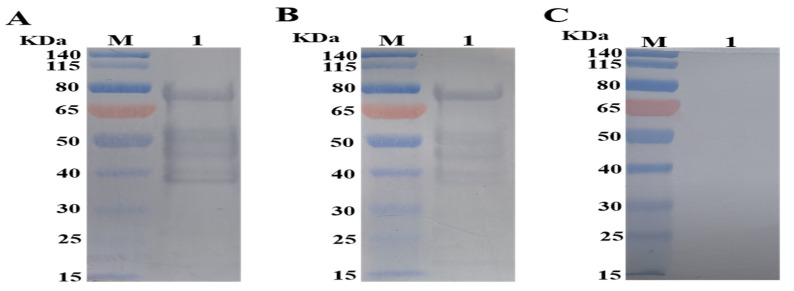
Western blot analysis of rEtROP17 (original Western blot figures are in Figure S1). (**A**) rEtROP17 was probed with mouse anti-His tag monoclonal antibody. (**B**) rEtROP17 was probed with chicken anti-*E. tenella* serum. (**C**) rEtROP17 was probed with normal chicken serum.

The identification of the purified protein was performed by Western blot using mouse anti-His tag antibody and chicken anti-*E. tenella* serum, respectively. Western blot analysis using mouse anti-His tag antibody as the primary antibody showed a single immunoreactive band with a relative molecular weight of approximately 78 KDa, which is consistent with its deduced size (Figure 2A). Meanwhile, the rEtROP17 could react with chicken anti-*E. tenella* serum (Figure 2B) but showed no reactivity with normal chicken serum (Figure 2C).

### 3.3. Expression Analysis of Native EtROP17 in Merozoites

**Figure 3:**
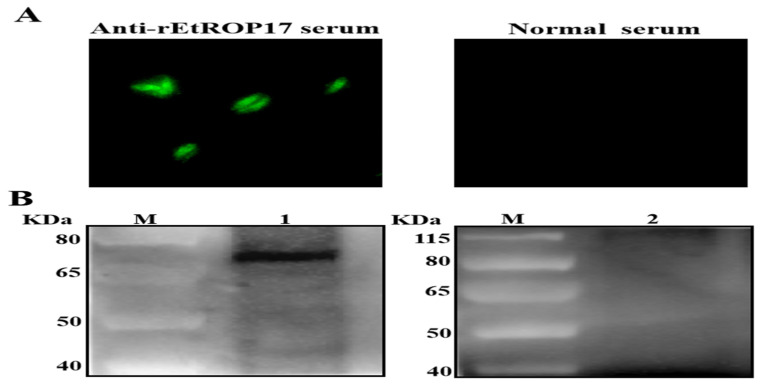
(**A**) Immunofluorescent staining of *E. tenella* merozoites, using mouse anti-rEtROP17 serum and normal mouse serum, respectively. (**B**) Lane M: protein marker; lanes 1–2: Western blot analysis of protein extracts of merozoites using mouse anti-rEtROP17 serum and normal mouse serum, respectively (original Western blot figures are in Figure S2).

The expression of EtROP17 in merozoites of *E. tenella* was determined using an indirect immunofluorescence technique. Merozoites were clearly labeled by mouse anti-rEtROP17 serum (Figure 3A). Control reactions using normal mouse serum as the primary antibody showed no staining (Figure 3A).

The expression of EtROP17 in merozoites was also detected through Western blot. Using mouse anti-rEtROP17 serum as the primary antibody, a unique band of approximately 71 KDa was observed (Figure 3B). The relative molecular weight was consistent with its deduced size. In contrast, no band was observed in the control group (Figure 3B).

### 3.4. IgY Titers and Protective Efficacy of Vaccination on E. tenella Challenge

**Figure 4:**
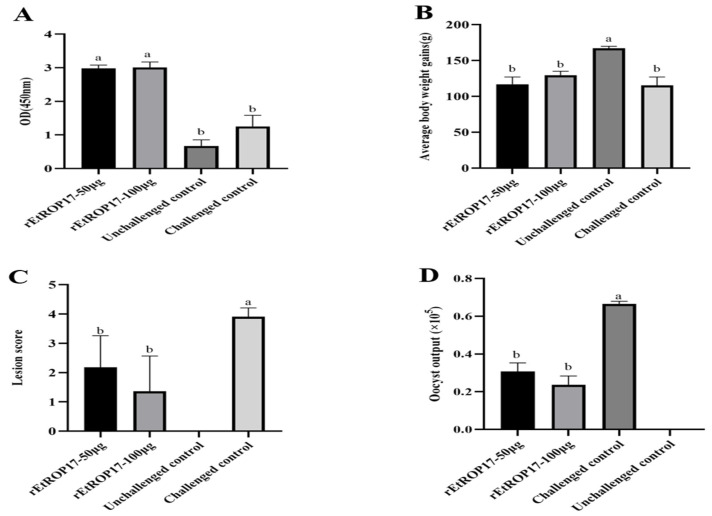
(**A**) IgY titers. (**B**) Relative body weight gain. (**C**) Cecal lesion scores. (**D**) Oocyst output. Bars sharing the same letters indicate no significant difference.

The IgY titers of vaccinated chickens were significantly higher than those of the challenged control and the unchallenged control groups (*p* < 0.05) (Figure 4A). Resistance to coccidiosis challenge infection was measured by body weight gain, cecal lesion scores, and oocyst output (Table 2). The body weight gain of group rEtROP17-100 μg was the highest, except for the unchallenged control group. However, no significant differences were observed between the immunized groups (50 μg or 100 μg of rEtROP17) and the challenged control group, and the body weight gain of group rEtROP17-50 μg was similar to that of the challenged control group. (Figure 4B). Cecal lesion scores in chickens immunized with 50 μg or 100 μg of rEtROP17 were significantly lower than those of the challenged control (*p* < 0.05) (Figure 4C). Chickens vaccinated with rEtROP17 showed a significant increase in oocyst output compared with the challenged control (*p* < 0.05) (Figure 4D).

## 4. Discussion

In the present study, we cloned and characterized the ROP17 of *E. tenella.* Sequencing analysis revealed that a single base difference at sequence position 1901 of the ROP17 of the SD-01 strain was observed compared with that of the Houghton strain. The results shown are representative of three independent experiments using high-fidelity DNA polymerase and different cDNA. *E. tenella* and *E. necatrix* are considered to be highly pathogenic species [21,22]. Intriguingly, the sequence similarity of ROP17 between *E. tenella* and *E. necatrix* is high, whereas the ROP17 of *E. tenella*, *E. maxima*, *E. acervulina*, and *E. mitis* share low sequence similarity [11]. Hence, ROP17 may represent a virulent factor.

*E. tenella* has a complex life cycle, and the endogenous stage includes the replication of parasites within chicken intestinal epithelial cells [23,24]. The asexual replicative phases are deemed to be responsible for the most damage to intestinal tissues [25]. Previous studies have shown that *Toxoplasma gondii* ROP17 are vital for survival within host cells [26,27]. The identification of genes expressed in the life cycle of *Eimeria* species is important for understanding their developmental biology, and the present study primarily aimed to determine the expression of EtROP17 in *E. tenella* merozoites. The expression of EtROP17 in *E. tenella* merozoites was confirmed by immunofluorescent staining as well as Western blot analysis. To the best our knowledge, this is the first report that EtROP17 is expressed in *E. tenella* merozoites, which is expected to lay a foundation for further functional studies.

In addition to cell-mediated immunity playing a decisive role in protection against infection, antibodies do exert an effective role in immunity [28]. Western blot analysis suggested that EtROP17 could be recognized by the serum of chickens experimentally infected with *E. tenella*. This indicated that EtROP17 could be recognized by the chicken immune system and induce an antibody response. This was further confirmed by the subsequent assay in the present study, which showed that chickens immunized with rEtROP17 had a significant increase in IgY titers compared with those of the challenged control and the unchallenged control groups. No significant differences between the challenged and the unchallenged groups in IgY titer were observed, which was consistent with the results of a previous study [29].

Our findings showed that vaccination with rEtROP17 was able to significantly decrease the oocyst output and alleviate cecal lesions as compared to the challenge control group. This demonstrated that EtROP17 might be an effective vaccine candidate against *E. tenella*. However, further studies based on larger numbers of *E. tenella* field isolates from different geographical areas are needed to determine genetic heterogeneity. Meanwhile, it is necessary to evaluate the protective efficacy of EtROP17 showing amino acid changes, as genetic polymorphisms of vaccine candidates may affect protective efficacy [30].

## 5. Conclusions

The present study identified and characterized EtROP17. Sequence analysis showed that a single base difference at position 1901 of the ROP17 of the SD-01 strain was observed compared with that of the Houghton strain. EtROP17 was expressed in the merozoite stage of *E. tenella* and may serve as a potential candidate for developing vaccines against *E. tenella*.

## Figures and Tables

**Table 1:** Experimental design of immunizations and challenges.

Groups	Delivery Route	At 7 Days of Age	At 14 Days of Age	Challenge ^1^
Unchallenged control	Subcutaneous injection	B buffer + ISA 71	B buffer + ISA 71	Unchallenged
Challenged control	Subcutaneous injection	B buffer + ISA 71	B buffer + ISA 71	Challenged
rEtROP17-50 μg	Subcutaneous injection	50 μg rEtROP17+ ISA 71	50 μg rEtROP17+ ISA 71	Challenged
rEtROP17-100 μg	Subcutaneous injection	100 μg rEtROP17+ ISA 71	100 μg rEtROP17+ ISA 71	Challenged

^1^ At 21 days of age, chickens, except for those in the unchallenged control group, were challenged orally with 1 × 10^4^ sporulated oocysts of *E. tenella*.

**Table 2:** Protective effects in each group.

Groups	Average Body Weight Gains (g)	Relative Body Weight Gain Rate (%)	Lesion Scores	Oocyst Output (×10^5^)
Unchallenged control	167.32 ± 9.19 ^a^	100	0 ^c^	0 ^c^
Challenged control	115.35 ± 40.89 ^b^	69.95	3.91 ± 0.3 ^a^	0.6665 ± 0.019 ^a^
rEtROP17-50 μg	116.97 ± 34.91 ^b^	74.68	2.18 ± 1.08 ^b^	0.3075 ± 0.064 ^b^
rEtROP17-100 μg	129.63 ± 19.03 ^b^	84.14	1.36 ± 1.21 ^b^	0.238 ± 0.065 ^b^

^a^, ^b^, ^c^: different letters denote statistically significant differences.

## Data Availability

Data supporting reported results will be provided upon request.

## Supplementary Materials

The following supporting information can be downloaded at: https://www.mdpi.com/article/10.3390/ani12050556/s1, Figure S1: Original Western blot figures of Figure 2; Figure S2: Original Western blot figures of Figure 3.
