# Supplementary material for: Identification and Protective Efficacy of Eimeria tenella Rhoptry Kinase Family Protein 17
Source: Animals (Basel). 2022 Feb 23;12(5):556. doi: 10.3390/ani12050556 (PMC8908856; doi:10.3390/ani12050556)
Supplement: Supplementary file 1 [file animals-12-00556-s001.zip › animals-1552756-supplementary.pdf]

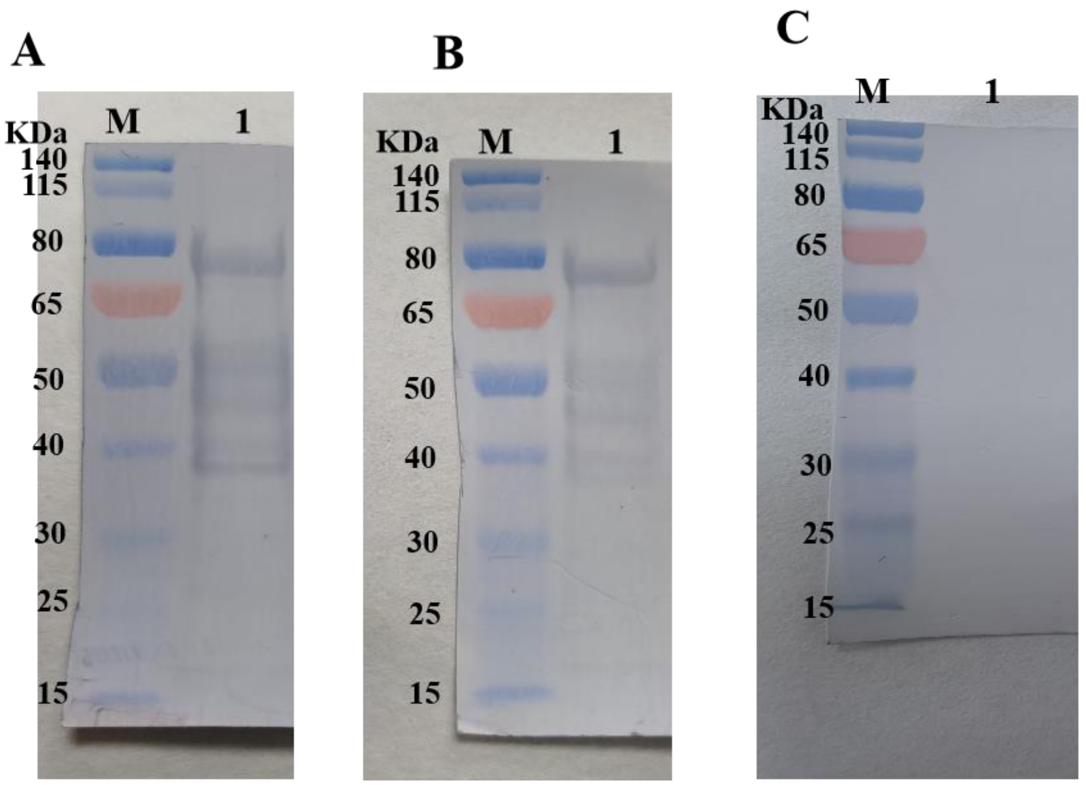

Figure S1. Original Western blot figures of Figure 2. (**A**) rEtROP17 was probed with mouse anti-His tag monoclonal antibody. (**B**) rEtROP17 was probed with chicken anti-*E. tenella* serum. (**C**) rEtROP17 was probed with normal chicken serum.

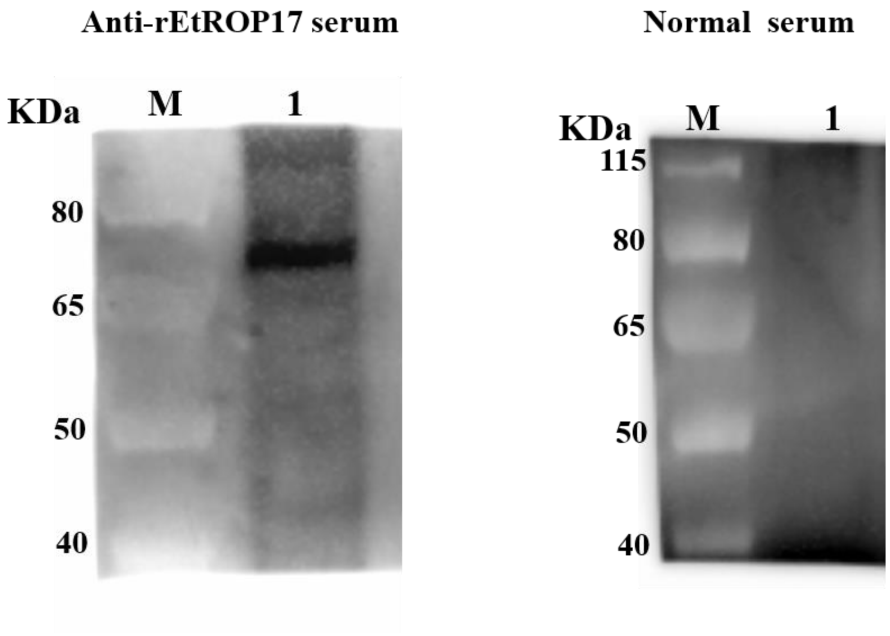

Figure S2. Original Western blot figures of Figure 3. Western blot analysis of protein extracts of merozoites, using mouse anti-rEtROP17 serum and normal mouse serum, respectively.
